# Supplementary material for: Rational Generation of Monoclonal Antibodies Selective for Pathogenic Forms of Alpha-Synuclein
Source: Biomedicines. 2022 Sep 2;10(9):2168. doi: 10.3390/biomedicines10092168 (PMC9496384; doi:10.3390/biomedicines10092168)
Supplement: Supplementary file 1 [file biomedicines-10-02168-s001.zip › biomedicines-1875402-supplementary.pdf]

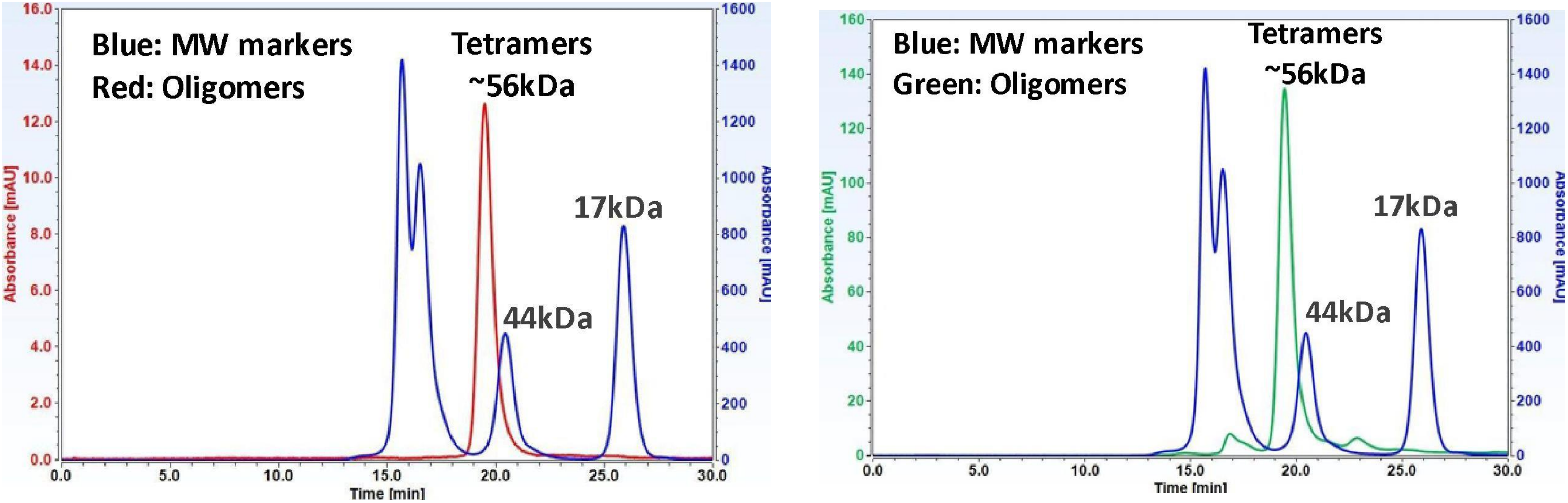

Supplemental Figure S1. SEC of oligomer preparations with demonstrated *in vitro* toxicity shows predominance of tetramers.

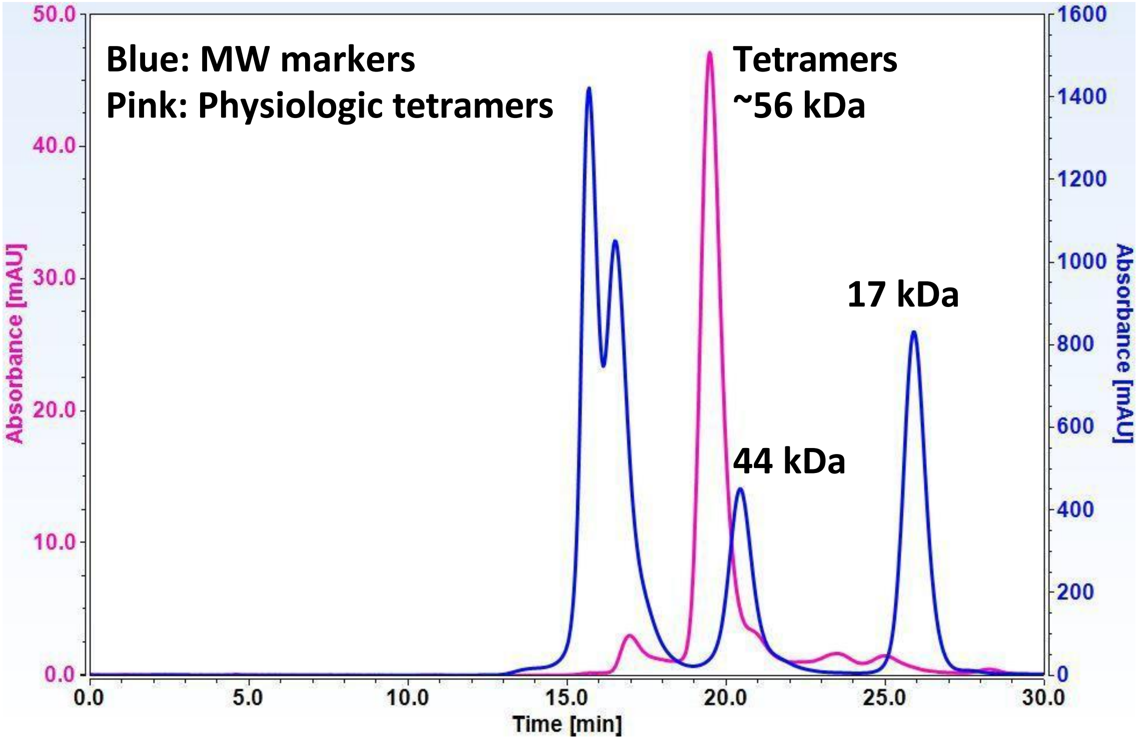

Supplemental Figure S2. SEC of physiologic tetramer preparations shows predominance of tetramers.

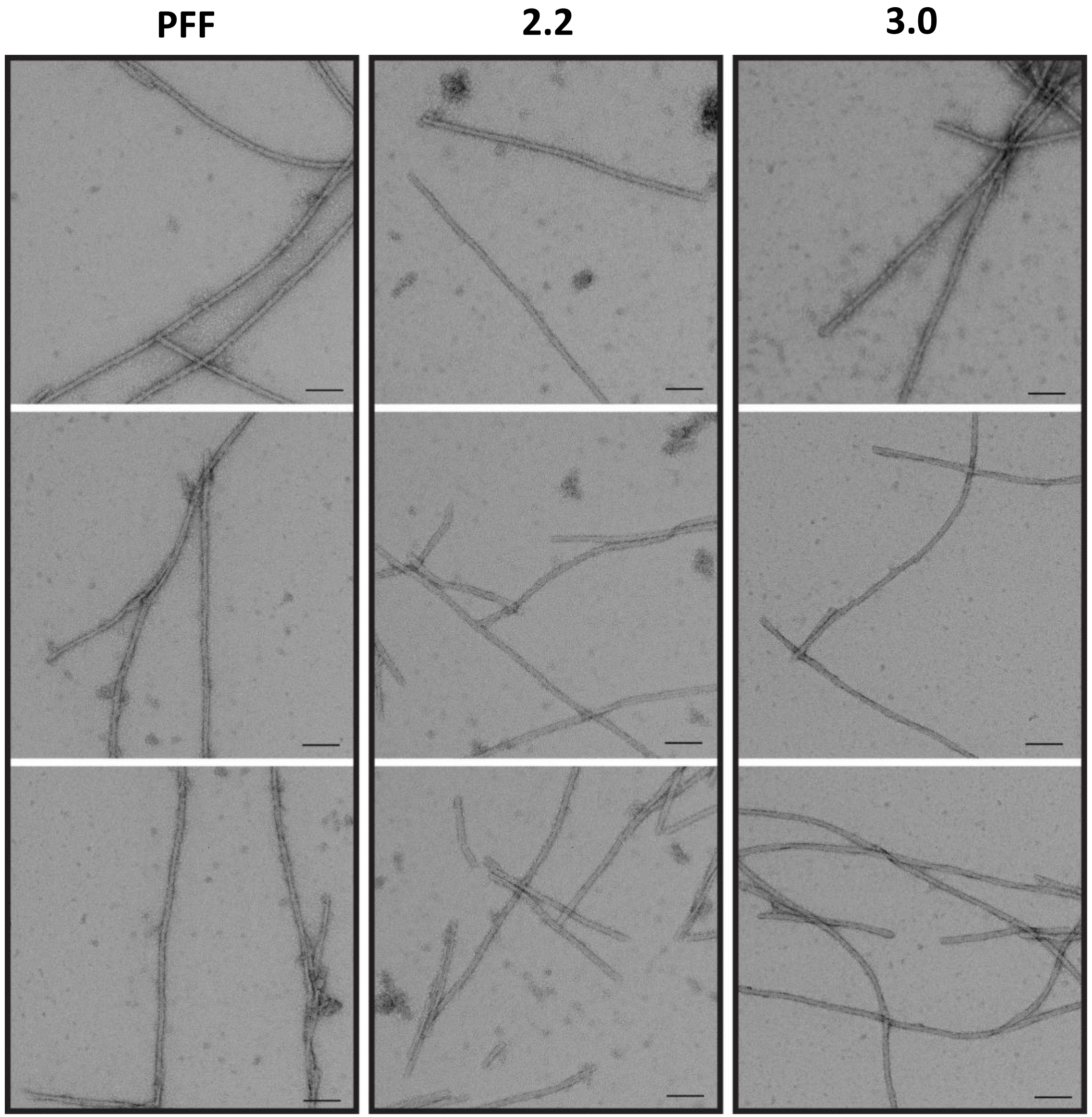

Supplemental Figure S3. Electron microscopic analysis of α-syn fibrils. Representative images of the negative stained fibril specimens acquired at 45,000x using a Ceta CMOS camera on a Talos L120C transmission electron microscope. The overall morphology and widths of the fibrils seeded by peptide scaffolds resemble that of fibrils seeded by PFFs. Scaffold 2.2 = cyclo(CGGKTKEGG), scaffold 3.0 = cyclo(CGGEKTKGGG). Scale bar = 100 nm.
